# Proposition of a novel animal model of systemic sclerosis induced by type V collagen in C57BL/6 mice that reproduces fibrosis, vasculopathy and autoimmunity

**DOI:** 10.1186/s13075-019-2052-2

**Published:** 2019-12-11

**Authors:** Walcy Rosolia Teodoro, Zelita Aparecida de Jesus Queiroz, Lais Araujo dos Santos, Sergio Catanozi, Antonio dos Santos Filho, Cleonice Bueno, Margarete B. G. Vendramini, Sandra de Morais Fernezlian, Esmeralda M. Eher, Percival D. Sampaio-Barros, Sandra Gofinet Pasoto, Fernanda Degobbi T. Q. S. Lopes, Ana Paula Pereira Velosa, Vera Luiza Capelozzi

**Affiliations:** 1 Rheumatology Division of the Hospital das Clinicas da Faculdade de Medicina da Universidade de Sao Paulo, FMUSP, São Paulo, SP, BR, Av. Dr. Arnaldo, 455, sala 3124, Cerqueira César, São Paulo, SP 01246-903 Brazil; 2 Lipid Laboratory of the Endocrinology and Metabology Discipline of the Hospital das Clinicas da Faculdade de Medicina da Universidade de Sao Paulo, FMUSP, Sao Paulo, SP Brazil; 3 Department of Pathology of the Hospital das Clinicas da Faculdade de Medicina da Universidade de Sao Paulo, FMUSP, Sao Paulo, SP Brazil; 4 Experimental Therapy Laboratory of the Department of Clinical Medicine of the Hospital das Clinicas da Faculdade de Medicina da Universidade de Sao Paulo, FMUSP, Sao Paulo, SP Brazil

**Keywords:** Type V collagen, Systemic sclerosis, Mouse model, Vascular, Fibrosis, Autoantibodies

## Abstract

**Background:**

Type V collagen (Col V) has the potential to become an autoantigen and has been associated with the pathogenesis of systemic sclerosis (SSc). We characterized serological, functional, and histopathological features of the skin and lung in a novel SSc murine model induced by Col V immunization.

**Methods:**

Female C57BL/6 mice (*n* = 19, IMU-COLV) were subcutaneously immunized with two doses of Col V (125 μg) emulsified in complete Freund adjuvant, followed by two intramuscular boosters. The control group (*n* = 19) did not receive Col V. After 120 days, we examined the respiratory mechanics, serum autoantibodies, and vascular manifestations of the mice. The skin and lung inflammatory processes and the collagen gene/protein expressions were analyzed.

**Results:**

Vascular manifestations were characterized by endothelial cell activity and apoptosis, as shown by the increased expression of VEGF, endothelin-1, and caspase-3 in endothelial cells. The IMU-COLV mice presented with increased tissue elastance and a nonspecific interstitial pneumonia (NSIP) histologic pattern in the lung, combined with the thickening of the small and medium intrapulmonary arteries, increased Col V fibers, and increased COL1A1, COL1A2, COL3A1, COL5A1, and COL5A2 gene expression. The skin of the IMU-COLV mice showed thickness, epidermal rectification, decreased papillary dermis, atrophied appendages, and increased collagen, COL5A1, and COL5A2 gene expression. Anti-collagen III and IV and ANA antibodies were detected in the sera of the IMU-COLV mice.

**Conclusion:**

We demonstrated that cutaneous, vascular, and pulmonary remodeling are mimicked in the Col V-induced SSc mouse model, which thus represents a suitable preclinical model to study the mechanisms and therapeutic approaches for SSc.

## Background

Systemic sclerosis (SSc) is a chronic, multisystem connective tissue disorder affecting the skin and the lung [1, 2]. The leading cause of death in SSc patients is pulmonary dysfunction as a result of interstitial fibrosis and vasculopathy [3, 4]. Endothelial cell injury, apoptosis, and progressive proliferation of the intima leads to vessel narrowing, hypoperfusion, and hypoxia, which subsequently could trigger skin and lung fibrosis, thus contributing to organ dysfunction and significant morbidity and mortality [5].

The advances in the understanding of the complex pathogenesis of SSc is primarily owing to the experimental data analyzing the mechanisms and biomarkers of the skin, vascular, and lung damage in SSc [6–8]. Mouse models are powerful tools to explore the pathogenesis of human autoimmune diseases [9] and can be categorized into the following two groups: *inducible models*, in which the disease is induced as for example by immunization, after which the transfer of autoimmune components or environmental factors occurs [10], and *spontaneous models*, where mice develop the disease due to genetic mutations or modifications without any further experimental manipulation [11]. Our first studies in animal models of SSc were performed in rabbits and showed vascular remodeling and fibrosis in the skin and lungs and immune reactivity after immunization with type V collagen (Col V) [12, 13]. However, due to handling limitations, high cost, and reproducibility, we considered that a mouse model would be a more viable and reproducible model for studying the pathogenic mechanisms of SSc. The capacity of induction of pulmonary fibrosis in previous models using C57BL/6 mice was the main reason for the selection of these mice lineages.

There are evidences indicating a role for type V collagen in the pathogenesis of SSc. It is established that the Col V isoform [α1(V)_2_, α2(V)] is ubiquitous in the tissues and is located within heterotypic collagen I/III/V fibrils [14, 15] and can be considered a sequestered antigen and has the potential to become an autoantigen when exposed to the immune system in several pathologies [16–20]. We demonstrated increased expression of Col V in the skin of patients in the early stages of SSc, correlating the anomalous deposit of this protein in the skin with the cutaneous thickening and disease activity [21]. Furthermore, we found that increased mRNA expression of Col V gene and abnormal Col V deposition in pulmonary tissue and lung fibroblast were correlated with fibrosis and a worsened pulmonary function in SSc patients [22, 23]. Interestingly, the nasal tolerance induction with Col V in a rabbit SSc model showed a decrease in the fibrotic process in the lung and skin and in the levels of fibrogenic cytokines, reinforcing the participation of Col V in autoimmunity and the pathogenesis of SSc [24].

Considering these aspects, in the present study, our aim was to characterize the serological and vascular manifestations and the functional and histopathological features of the skin and lung to establish a novel SSc murine model induced by Col V immunization.

## Materials and methods

### Experimental model

C57BL/6 female mice (*n* = 38), 6–7 weeks old, were provided from the animal facility of our institution and maintained in specific pathogen-free conditions with free access to food and fresh water in a temperature-controlled room (22–24 °C) on a 12-h light/dark cycle. All of the experimental procedures were performed according to The Committee on Ethical Use of Laboratory Animals of Faculty of Medicine at the University of São Paulo (Process code: 170/15).

**Fig. 1:**
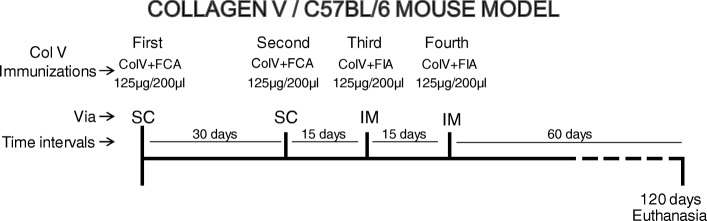
Experimental design. The scheme shows the immunization protocol performed on C57BL/6 mice with type V collagen, detailing the dose, the period of inoculation of the antigen, and euthanasia of animals. FCA Freund’s complete adjuvant, FIA Freund’s incomplete adjuvant, SC subcutaneous, IM intramuscular

The C57BL/6 mice (*n* = 19; IMU-COLV) were immunized with subcutaneous injections of 125 μg/200 μl of human placental Col V (Sigma) that was diluted in 10 mM acetic acid and emulsified with an equal volume of complete Freund’s adjuvant. Four weeks later, an identical dose was administered, and two other boosters were given at 15 days’ intervals (125 μg/200 μl Col V plus incomplete Freund’s adjuvant) by intramuscular injection (Fig. 1). The control group (*n* = 19; CT) was inoculated with 10 mM acetic acid plus complete or incomplete Freund’s adjuvant. All of the mice were maintained for 120 days after the first immunization.

### Specimen preparation

For the histologic, morphometric, and biochemical analyses of the IMU-COLV (*n* = 10) and CT (*n* = 9) groups, the animals were anesthetized by an intraperitoneal injection of ketamine hydrochloride (Ketalar) (100 mg/kg of body weight) and xylazine (Rompun) (10 mg/kg of body weight) and euthanized by nuchal dislocation. The right lung and skin samples were immersed in buffered formalin 10% for staining with hematoxylin-eosin (H&E), Masson Trichrome, and Picrosirius. The samples of the left lung were maintained at − 20 °C in physiological solution for the biochemical analysis and at − 196 °C for the molecular analysis.

### Respiratory mechanics evaluation

For the respiratory mechanics evaluation, the mice in the IMU-COLV (*n* = 9) and control (*n* = 10) groups were intraperitoneally anesthetized with thiopental (70 mg/kg), tracheostomized, and mechanically ventilated using a FlexiVent ventilator (Scireq, Montreal, QC, Canada). We used the forced oscillatory technique and a constant phase model [25] to obtain the airway resistance (Raw), tissue damping (Gtis), and tissue elastance (Htis) parameters [26].

### Immunofluorescence, immunohistochemistry, and histomorphometry

Lung and skin sections (3–4 μm) were dewaxed in xylol and hydrated in graded ethanol. Antigen retrieval was accomplished with an enzymatic treatment with porcine pepsin (10,000 dry unit/ml) (Sigma) in 0.5 N acetic acid for 30 min at 37 °C. Nonspecific sites were blocked with 5% skim milk in phosphate buffer saline (PBS) for 30 min. The specimens were incubated overnight at 4 °C with rabbit polyclonal anti-human Col I (1:700, Rockland), anti-human Col III (1:200, Rockland), and anti-human Col V (1,1.000, Rockland) antibodies diluted in PBS and stained with an ALEXA FLUOR 488 goat anti-rabbit IgG antibody (Invitrogen) diluted 1:200 in a PBS solution containing 0.006% Evans blue. For the negative and background fluorescence controls, sections were incubated with PBS instead of the specific antibody. Tissues were mounted in buffered glycerol, and images visualized in a fluorescence microscope (Olympus BX51). For the histomorphometric analysis, the images were processed with Image Pro-Plus 6.0 software [22]. The density of Col I, Col III, and Col V fibers was measured in the lung parenchyma and in the skin area in 10 randomly selected microscopic fields at a magnification of × 200. The threshold for collagen fibers was established for all slides after the contrast was enhanced to the point at which the fibers were easily identified as green bands. The density of the collagen fibers was expressed as the ratio between the measured fibers divided by the total area studied × 100 [22]. All of the microscopic fields for each slide were quantified, and the results were expressed as the means of these fields.

For the immunohistochemistry analysis, the lung sections were submitted to a high temperature (121 °C) for 1 min for antigen retrieval. After blocking of nonspecific sites with 3% hydrogen peroxide for 5 min, the specimens were stained with anti-caspase-3 (Santa Cruz Biotechnology; 1:100 dilution), anti-endothelin-1 (Santa Cruz Biotechnology; 1:50 dilution), and anti-vascular endothelial growth factor (VEGF) (Santa Cruz Biotechnology; 1:1000 dilution) antibodies. The reactions were stained with Vectastain ABC (Vector Laboratories). The color was developed with 3,3-diaminobenzidinetetrahydrochloride (Vector Laboratories) and counterstained with H&E. The isotype IgG was used as a negative control. To access uniform and proportional lung samples, 10 fields were randomly analyzed in the lung parenchyma at × 1000 magnification for caspase-3, endothelin-1, and VEGF expression, and the cells count was done by a manual point count in each field (Software Image Pro-Plus 6.0). The results show the percentage of positive cells in the lung parenchyma per micrometer square.

### Quantitative assessment of collagen content

The total lung collagen deposition was estimated by measuring the 4-hydroxyproline content as previously described with modifications [27]. Briefly, the lung portions were freeze-dried (Edwards, Modulyo), weighed, and hydrolyzed in 6 N HCl for 22 h at 110 °C. Lung hydroxyproline levels were determined spectrophotometrically by absorbance at 560 nm, and the results were expressed as nanograms of 4-hydroxyproline per milligram of protein [27].

### Quantitative real-time polymerase chain reaction (qRT-PCR)

Total RNA was isolated using Trizol reagent (Invitrogen), according to the manufacturer’s protocol. The expression levels of COL1A1 (collagen I α1 chain), COL1A2 (collagen I α2 chain), COL3A1 (collagen III α1 chain), COL5A1 (collagen V α1 chain), COL5A2 (collagen V α2 chain), CASP3 (caspase-3), VEGF, and ET-1 (endothelin-1) (Table 1) were evaluated by qRT-PCR. All qRT-PCR reaction mixtures were prepared using Superscript Platinum III one-step kits that had SYBR Green incorporated (Invitrogen). The cDNA production and amplification steps were performed on a Step One (Applied Biosystems) thermocycler with 100 ng of total RNA per sample. Relative expression was determined by the 2-ΔΔCT method using the housekeeping gene β-2 microglobulin (BETA-2) for normalization.

**Table 1:** Oligonucleotides employed for quantitative qRT-PCR

Gene	Sense 3′ → 5′	Antisense 5′ → 3′
COL1A1	GAG CGG AGA GTA CTG GAT CG	GCT TCT TTT CCT TGG GGT TC
COL1A2	CCG TGC TTC TCA GAA CAT CA	GAG CAG CCA TCG ACT AGG AC
COL3A1	GCA CAG CAG TCC AAC GTA GA	TCT CCA AAT GGG ATC TCT GG
COL5A1	GGT CCC TGA CAC ACC TCA GT	TGC TCC TCA GGA ACC TCT GT
COL5A2	CT CAG GGA ATT GAT GGA GA	AGA GCC AGG CAT GAG TCC TA
ET-1	CTG CCA AGC AGG AAA AGA AC	TTG TGC GTC AAC TTC TGG TC
CASP3	GGG CCT CTT GAA CTG AAA AA	CCG TCC TTT GAA TTT CTC CA
VEGF	AGC ACA GCA GAT GTG AAT GC	AAT GCT TTC TCC GCT CTG AA
BETA-2	CAT GGC TCG CTC GGT GAC C	AAT GTG AGG CGG GTG GAA CTG

### ELISA for anti-col I, III, IV, and V

Microtiter plates were coated with 5 μg/well of Col I, III, IV, and V (Sigma) in bicarbonate buffer, pH 9.6, and incubated overnight at 4 °C. After blockade with 1% BSA-PBS (Sigma) for 60 min, duplicates of the mouse serum samples (1:100) were diluted in 1% BSA-PBS with 0.05% Tween 20 and incubated for 60 min at room temperature, followed by incubation with goat-anti-mouse IgG conjugated alkaline phosphatase (Sigma). The reaction was revealed by adding 1 mg/ml of p-nitrophenyl phosphate (pNPP) as substrate diluted in 1 M diethanolamine and 0.5 mM MgCl_2_ in a pH 9.8 buffer. The optical density (OD) of the samples were read at 405 nm on a microplate reader (Labsystems Multiskan). The cutoff was determined based on the means plus 3 SD (standard deviation) of eight normal serum samples.

### Antinuclear antibodies

Antinuclear antibodies (ANA) were detected by indirect immunofluorescence using Hep2 cells, according to the International Consensus on ANA Patterns (ICAP) [28]. An ALEXA FLUOR 488-conjugated goat-anti-mouse IgG antibody (Invitrogen) at a 1:200 dilution was used as the secondary antibody. The slides were examined in an Olympus BX51 fluorescence microscope.

### Statistical analysis

Statistical comparisons were performed using GraphPad Prism 6 (GraphPad Software). The differences among groups were assessed by a one-way ANOVA followed by Tukey’s test. Data are expressed as the means ± standard error (SEM). A *p* value less than 0.05 was considered significant.

## Results

### Col V immunization induces lung and skin inflammation and fibrosis, mimicking human SSc

**Fig. 2:**
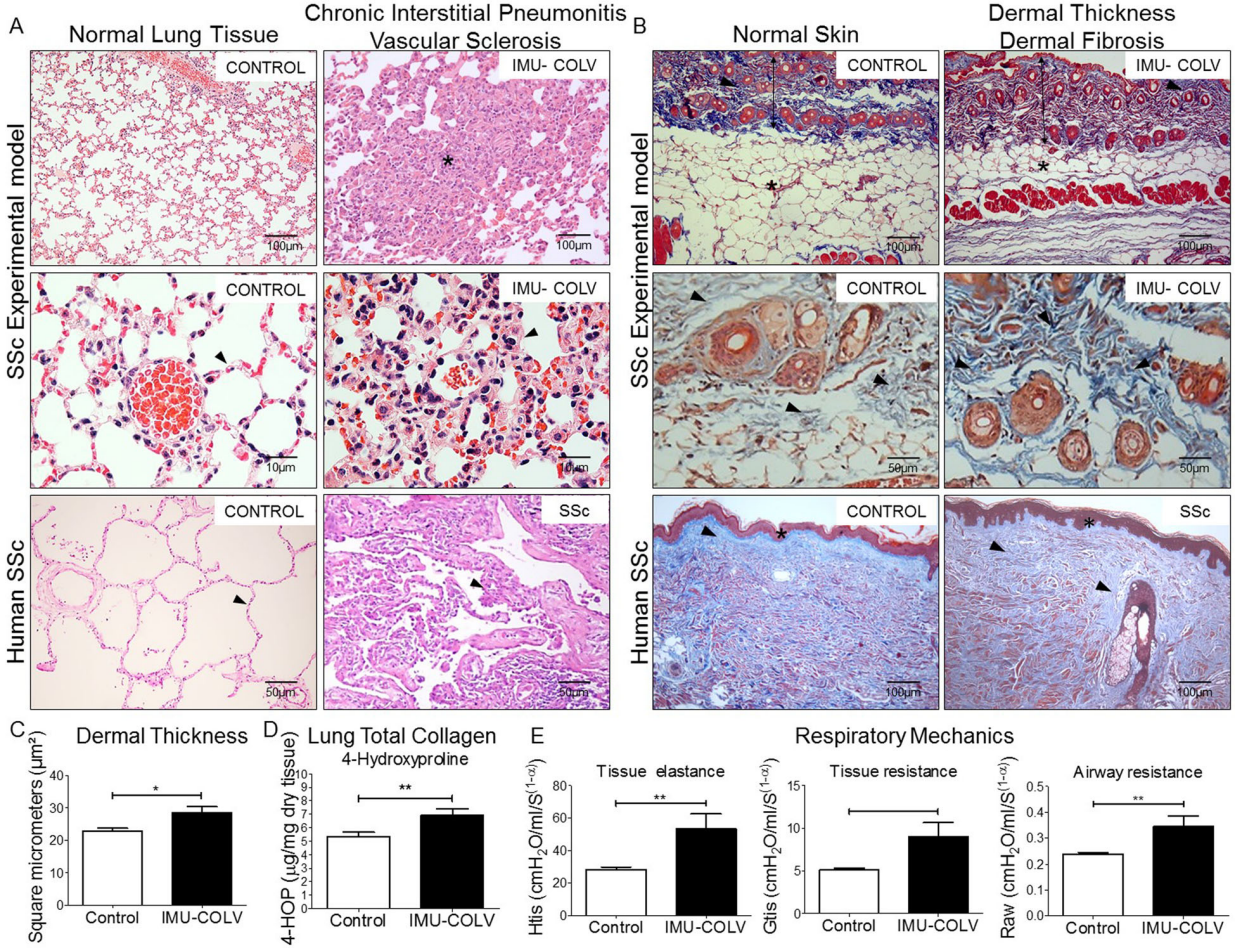
Changes in lung and skin morphology, dermal thickness, total lung collagen, and lung mechanics following the immunization with Col V in C57BL/6 mice and human SSc. C57BL/6 mice model was established by injection of Col V, as indicated as IMU-COLV, had similar changes to the traditional human SSc (**a**, **b**). The lung tissues were stained with H&E (**a**) and skin with Masson’s trichrome staining (**b**). Two independent individuals measured dermal thickness with Image Pro-Plus. Dermal thickness was shown to be significantly increased in Col V-immunized mice whereas control mice did not show an increase in dermal thickness (**c**). Lung total collagen content was measured with 4-hydroxyproline assay. Mice receiving Col V exhibited increased collagen content in the lung compared with control (**d**). Lung mechanics in mice immunized with Col V showed increased tissue elastance and resistance compared to control (**e**). Values are the means ± s.e (*n* = 38). *P* < 0.05

An examination of the lung parenchyma at 120 days showed striking changes in Col V immunized mice compared to the controls that received Freund’s adjuvant alone. We observed a homogeneous septal thickening through inflammation and fibrosis with the maintenance of the lung histoarchitecture, thus characterizing a nonspecific interstitial pneumonia (NSIP) histologic pattern. The intrapulmonary arteries from each specimen that was evaluated showed isolated hypertrophy of the media and fibrosis of the adventitia layer (Fig. 2a). To gain additional insight into the injury of Col V immunized mice, we also investigated the skin changes at the morphological level. Notably, marked changes were characterized by epidermal rectification, decreased papillary dermis, atrophy of the cutaneous appendages, and loss of adipocytes within of the cutaneous compartment. The reticular and deep dermis, including blood vessels and cutaneous appendages, were thickened by collagen fibers, thus mimicking the histological pattern found in human scleroderma (Fig. 2b). Additionally, a quantitative analysis of skin thickness showed that the Col V immunized group showed increased skin thickening in relation to the control group (*p* = 0.0132) (Fig. 2c). The lung and skin morphology of the SSc patients and Col V immunized mice were very similar, as shown by a routine analysis of H&E-stained sections**.** Both groups showed homogeneous septal thickening and maintenance of the septal architecture, in addition to inflammation with fibrotic septal thickening. Vascular sclerosis was similar in both groups (Fig. 2a and b).

The hydroxyproline assay revealed a significant increase in the total collagen of lungs from Col V-immunized mice compared to control (*p* = 0.0071) (Fig. 2d). In agreement with the remodeling process that is characterized by inflammation, fibrosis, and an increase in the total collagen content, the lung mechanics exhibited a significant increase in tissue elastance (*p* = 0.0047) and airway resistance (*p* = 0.0022) in the immunized animals compared to the controls (Fig. 2e). The above findings provide clear evidence of an extensive parenchymal and vascular fibrosis in the lung and skin of Col V-immunized mice.

### Col V immunization regulates the remodeling and overexpression of genes/proteins and collagen synthesis

**Fig. 3:**
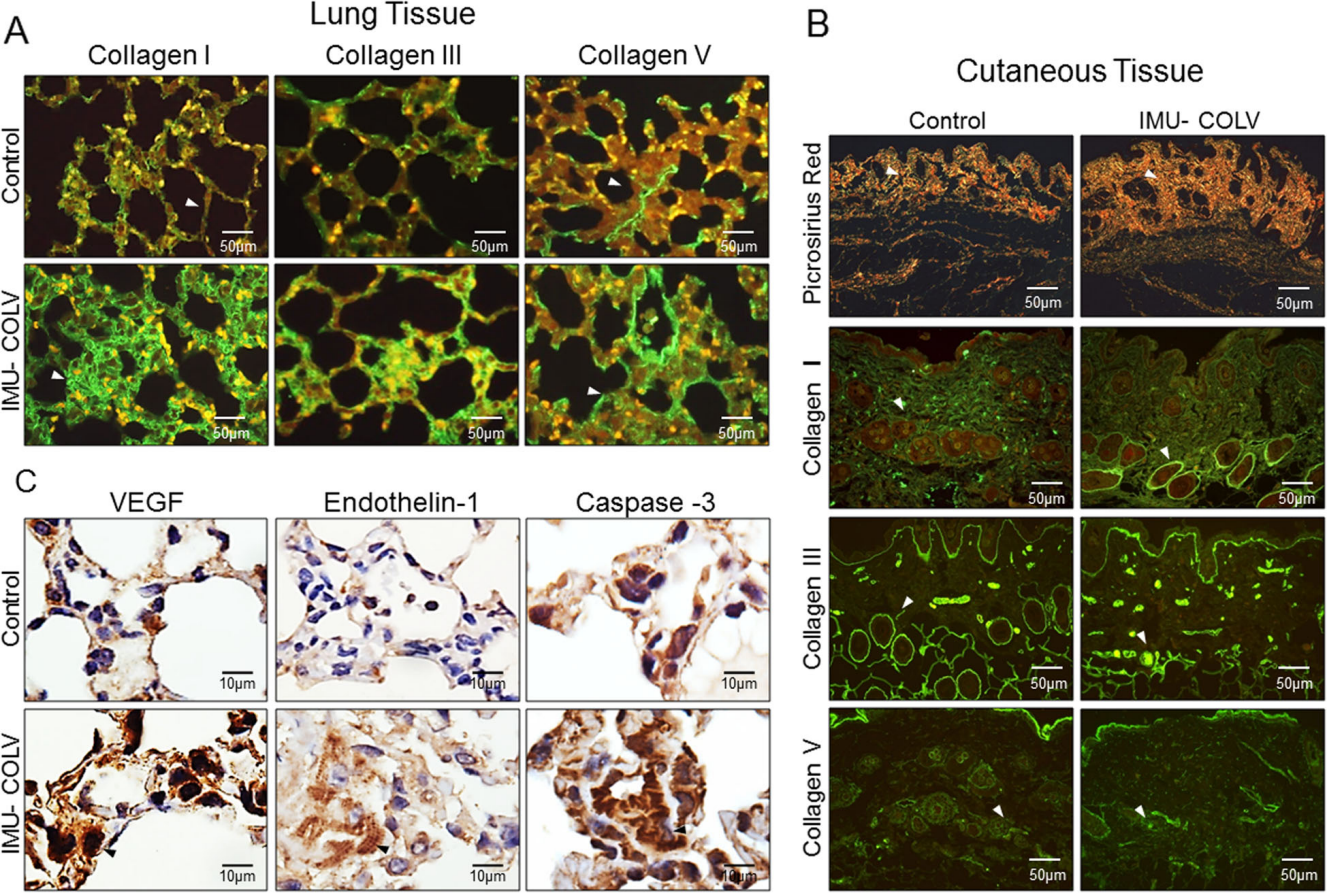
Changes in collagen fibers in the lung and skin lesions and immunohistochemical analysis of vascular injury in the lung following the Col V immunization in C57BL/6 mice. Immunofluorescence was performed to characterize collagen I (Col I), collagen III (Col III), and collagen V (Col V) in the lung (**a**) and skin (**b**). Mice receiving Col V exhibited increased deposition of Col I and Col V fibers in the lung and skin compared with control. The Picrosirius staining in skin samples showed total collagen in Col V-immunized mice and control (**b**). Immunohistochemical analysis was performed on the lung C57BL/6 mice receiving the treatment of Col V. Positive cells staining with endothelial markers VEGF and endothelin-1 and apoptosis marker caspase-3 were indicated by brown color (**c**)

**Fig. 4:**
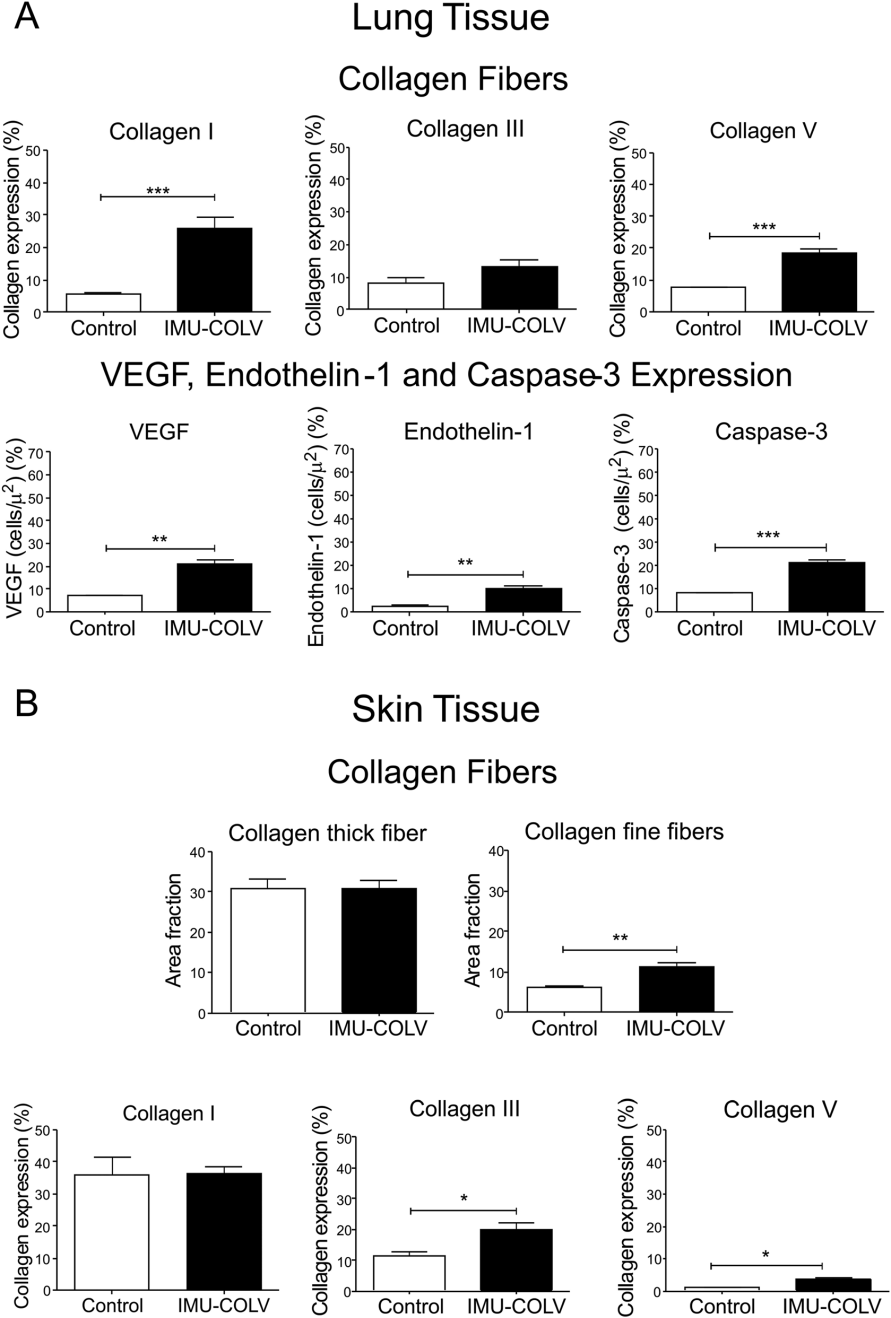
Increased collagen fibers density in the lung and skin and increase expression of endothelial markers of vascular injury in mice following the immunization with Col V. Quantification of collagen I (Col I), collagen III (Col III), and collagen V (Col V) and endothelial markers in the lung (**a**) and Col I, Col III, and Col V in the skin (**b**) by Image Pro-Plus of mice immunized with Col V. The quantification of the Picrosirius staining showed collagen fine fibers increase in Col V-immunized mice compared with control. Data are mean ± s.e. (*n* = 38) *P* < 0.05

The immunofluorescence analysis shows the expression of Col I, Col III, and Col V in the lung and skin of mice after 120 days of Col V immunization (Fig. 3a and b). In the lungs, the control mice exhibit Col I fibers that are loosely arranged along the basement membrane (BM), assume a uniform distribution, and enhance the alveolar architecture (Fig. 3a). In contrast, the significantly thicker type I collagen fibers are tightly packed along the BM, modifying the usual histoarchitecture of the alveoli when compared to that of the control mice (*p* = 0.0003) (Figs. 3a and 4 a). The alveolar septa in mice after 120 days of Col V immunization exhibits a significant increase of the thin Col III fibers, which are irregularly distributed along the BM and smaller vessels when compared to those of the control group (Figs. 3a and 4a). An analysis of Col V in the control group shows loose fibers in a homogeneous, linear distribution along the BM and around the vessels (Figs. 3a) that is consistent with normal lung architecture. In contrast, after 120 days, lungs from Col V immunized mice exhibit a significant increase in thick Col V fibers, which assume an irregular and micronodular distribution involving the BM and smaller vessels when compared to those of the control mice (*p* < 0.0001) (Figs. 3a 4a).

In the skin, the Picrosirius staining visualized under polarized light shows predominance of collagen fibers with yellow-orange birefringence (characteristic of thick fibers), arranged in a regular manner in the papillary dermis in the control group (Fig. 3b). In contrast, the Col V-immunized mice exhibit collagen fibers with yellow-orange birefringence, arranged irregularly in the cutaneous tissue when compared to the skin of control animals, as well as a mixture of collagen fibers with white-green birefringence (characteristic of fine fibers) (Fig. 3b). The histomorphometric analysis shows an increase of fine fibers of collagen in the skin from Col V immunized mice in relation to the control (*p* = 0.0073) (Fig. 4b). Confirming these data, the basement membrane and vessels from the skin of the Col V-immunized mice exhibit a significant increase in fine fibrillary Col III when compared to those of the control group (*p* = 0.0147) (Figs. 3b and Fig. 4b). An analysis of Col V in the control groups shows loose fibers in a homogeneous, linear distribution along the BM and around the vessels and cutaneous appendages (Fig. 3b) that is consistent with normal skin architecture. In contrast, after 120 days, the skin from the Col V-immunized mice exhibits a significant increase in the thick Col V fibers, which assume an irregular distribution around the BM and smaller vessels when compared to those of the control group (*p* = 0.0111) (Figs. 3b and 4b). In addition, the control and Col V-immunized mice exhibit similar amounts of Col I fibers, with a homogeneous pattern in the papillary and reticular dermis regions. Col I is most evident in the BM and around the vessels and cutaneous appendages (Figs. 3b and 4b).

**Fig. 5:**
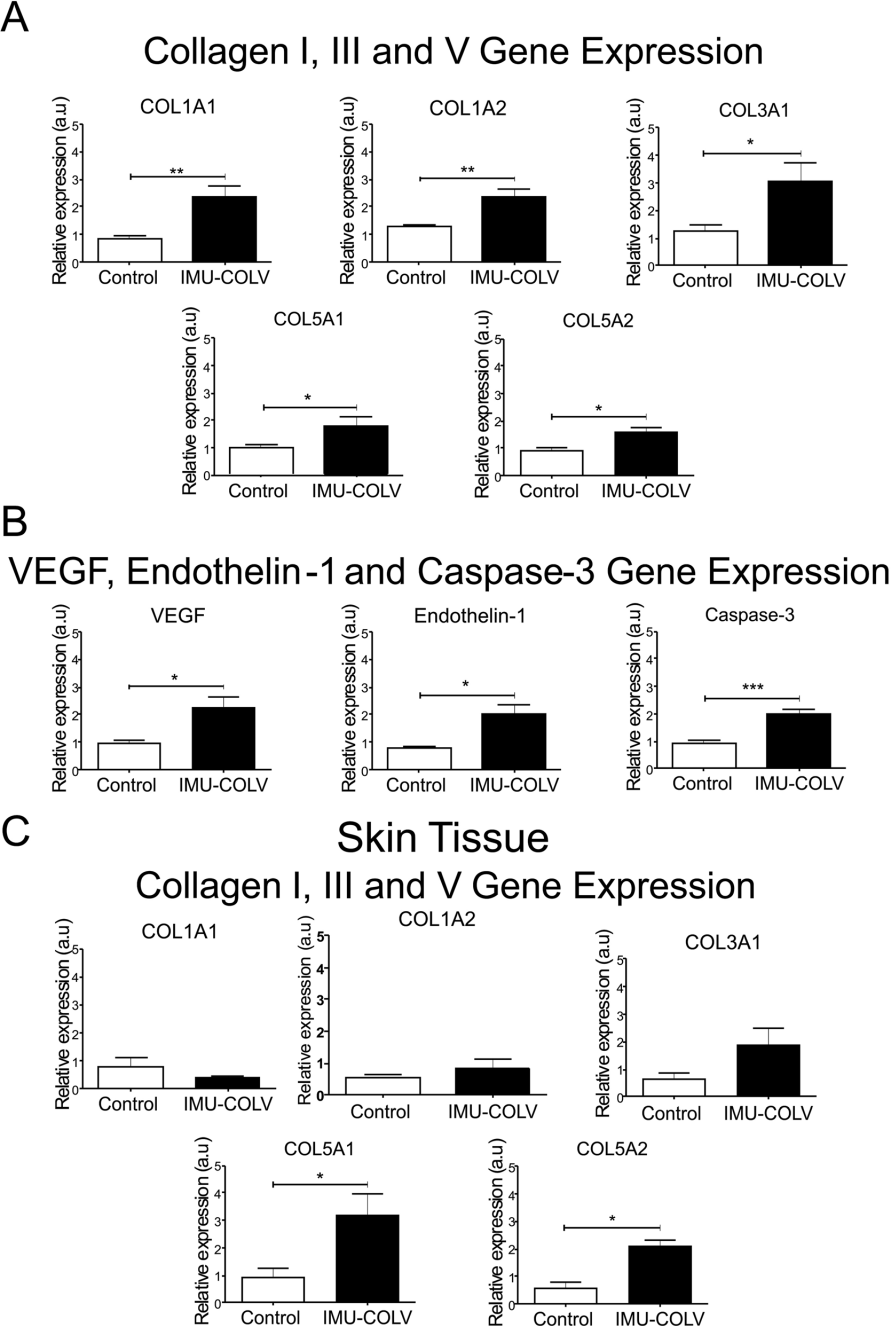
Quantitative qRT-PCR analysis of collagen I (COL1A1 and COL1A2), collagen III (COL3A1) and collagen V (COL5A1 and COL5A2) genes expression in the lung (**a**) and skin (**c**) tissues, and endothelial expression of VEGF, Endothelin-1 and Caspase-3 in lung tissue (**b**) from C57BL/6 mice immunized with Col V. Data represent mean ± s.e (*n* = 38) *P* < 0.05

Additionally, qRT-PCR was performed on the whole lung and skin mRNA that was collected from the control and Col V-immunized mice when they were euthanized at 120 days. Consistent with the quantification of the total collagen content (Fig. 2d), Col I, Col III, and Col V (Fig. 4a) in the lung, the COL1A1 (*p* = 0.0070), and COL1A2 (*p* = 0.0016) genes, which encode the α chains of Col I (Fig. 5a), the COL3A1 (*p* = 0.0350) gene, which encodes the α1 chain of Col III (Fig. 5a), and the COL5A1 (*p* = 0.0469) and COL5A2 (*p* = 0.0252) genes, which encode the Col V α1 and α2 chains, respectively, were significantly increased during the fibrotic response in Col V-immunized mice compared to the control group (Fig. 5a). The gene expression in the skin mRNA samples shows increase expression of COL5A1 (*p* = 0.0316) and COL5A2 (*p* = 0.0401) genes, respectively, of the Col V α1 and α2 chains in Col V-immunized mice compared to the control (Fig. 5c).

### Col V immunization promotes gene/protein overexpression and endothelial activity

We also performed immunohistochemistry and qRT-PCR on whole lung mRNA samples from the control and Col V-immunized mice that were euthanized at 120 days to detect endothelial activity and apoptosis. Immunostaining showed strong signals for ET-1, VEGF, and caspase-3 in the endothelial cells of the Col V immunized mice when compared to those of the control group (Fig. 3c). Apoptotic endothelial cells, as shown by increased caspase-3, were frequently found in the lung vessels of Col V-immunized mice when compared with those of the controls (Fig. 3c), mainly in regions with important activity and remodeling. In fact, the apoptotic index was significantly higher in the endothelial cells of the Col V-immunized mouse model when compared with the healthy group (*p* = 0.0007) (Fig. 4a). When stained by immunohistochemistry, the ET-1+ and VEGF+ endothelium in the vascular areas of pulmonary interstitium in the normal and Col V-immunized mice appears as brownish cells. Numerous cells in the vascular areas of interstitial pneumonia of the Col V-immunized mice expressed increased ET-1 (*p* = 0.0022) and VEGF (*p* = 0.0040) when compared to healthy controls (Figs. 3c and 4a). A quantitative analysis of the proteins confirmed that the morphologic distributions and the gene expression levels of ET-1 (*p* = 0.0351), VEGF (*p* = 0.0019), and caspase-3 (*p* = 0.0001), which were significantly increased in the vascular areas of Col V-immunized animals when compared to those of healthy controls (Fig. 5b).

### Col V immunization induces circulating autoantibodies

**Fig. 6:**
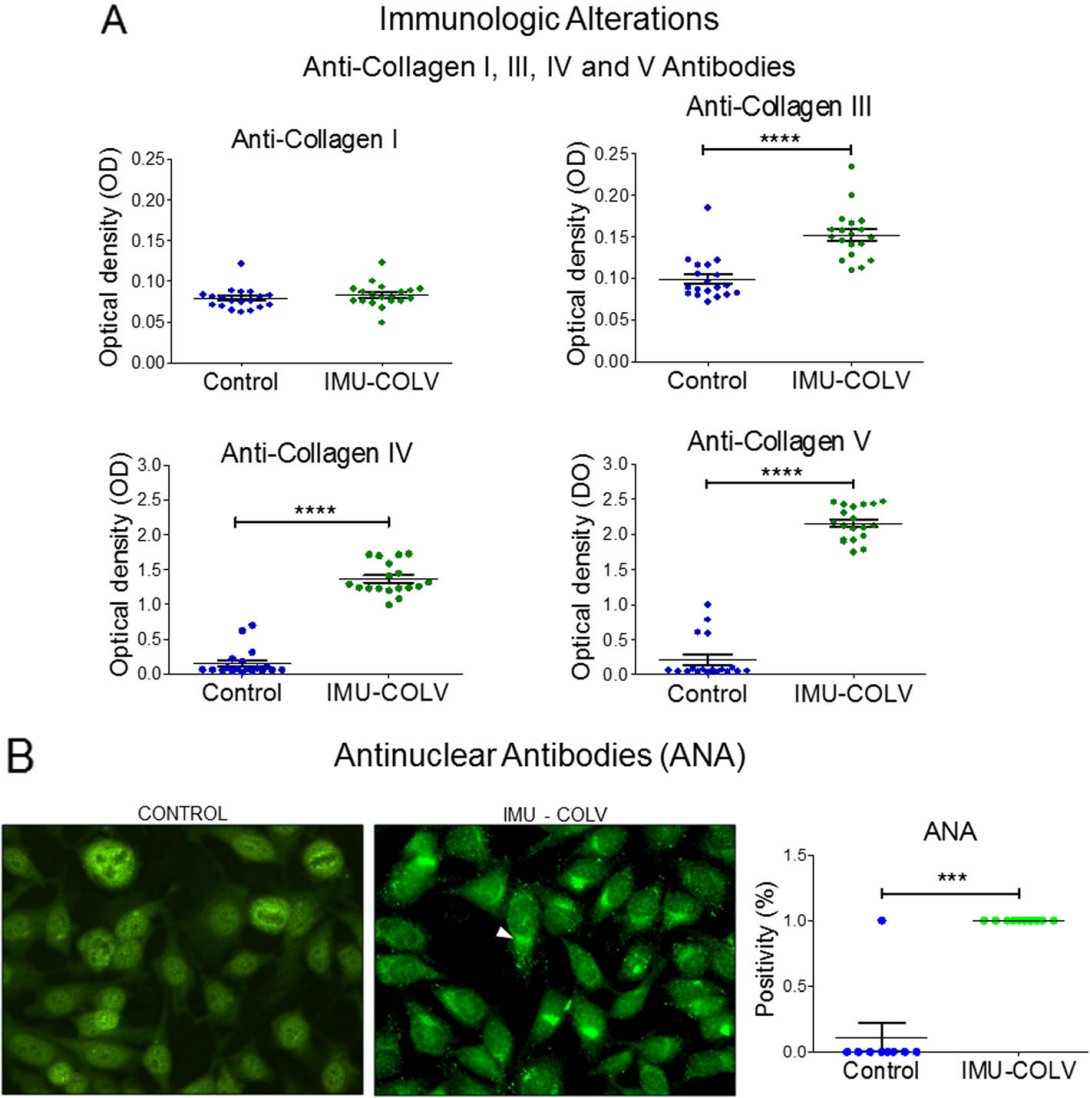
ELISA assays showed elevated anti-collagen III, IV, and V antibodies (**a**) and immunofluorescence assays showed a higher levels of antinuclear antibodies (ANA) (**b**) in the serum from the mice immunized with Col V. The immunofluorescence shows ANA with a fine dotted and/or Golgi cytoplasmic pattern in serum from C57BL/6 mice immunized with Col V (**b**, arrow). Data represent the mean ± s.e. (*n* = 38). *P* < 0.05

Confirming our previous reports, the Col V-immunized mice developed multiorgan fibrosis by day 120 that was identified by H&E staining in the skin and lung samples (Fig. 2a). At this time point, the immunized mice also developed antinuclear and anti-collagen antibodies (Fig. 6). Therefore, 92% of animals in the Col V-immunized group presented positive ANA in a fine dotted and/or Golgi cytoplasmic pattern (Fig. 6b). In contrast, this ANA pattern was not observed in the animals from the control group (*p* = 0.001) (Fig. 6b).

In addition, all of the Col V-immunized mice presented with significant increases in antibodies to Col V (*p* < 0.0001) (Fig. 6a). Interestingly, an unexpected increased positivity of antibodies to Col IV was observed in the Col V-immunized mice compared to control (*p* < 0.0001) (Fig. 6a). Although both groups presented with low titers of antibodies to Col III, a significant increased positivity was found in the Col V-immunized mice when compared to the control group (*p* < 0.0001) (Fig. 6a). The levels of positivity of antibodies to Col I were similar between the two groups (Fig. 6a).

## Discussion

Under the conditions of this study, we propose that C57BL/6 mice immunized with Col V can be considered a novel animal model that potentially contributes to the understanding of the links between inflammation, vascular injury, and fibrosis in SSc. This proposition is based on the main findings of this study: (1) fibrosis in the skin and lung; (2) abnormal lung mechanics; (3) overexpression of genes which encode chains of Col I, Col III, and Col V; (4) vasculopathy with strong signals for ET-1, VEGF, and caspase-3 and (5) autoimmunity with positive ANA, in addition to anti-Col III and anti-Col IV autoantibodies.

We selected C57BL/6 mice for our experiment because they represent an animal lineage very susceptible to develop pulmonary fibrosis, a manifestation linked to higher morbidity and mortality in SSc. In fact, pulmonary fibrosis was identified by the intense deposition of collagen in the vessels and interstitium, reproducing conditions similar to those observed in SSc patients [29, 30]. Of note, in Col V-immunized C57BL/6 mice, the presence of pulmonary vascular involvement was evident and characterized by inflammatory infiltrate in the lung tissue, endothelial apoptosis, and vascular remodeling. In this aspect, this new model can aggregate value to the study of the pathogenesis of SSc, as it can analyze the vascular and the fibrotic components of the disease, as well as the immunological reactivity. It took 4 years for the establishment of the lung-susceptible C57BL6 mouse model of SSc, after we have observed similar findings in a rabbit model [12, 13]. It is important to highlight that this new model can contribute to a better understanding of the earliest phases of skin tissue involvement and the evolution of the various stages of pulmonary fibrosis in SSc, as it is really difficult to obtain skin and lung samples from the SSc patients.

Clearly, Col V triggers a SSc model in mice, since it is a protein with peculiar biochemical characteristics and has great immunogenic potential [15, 31, 32]. In addition, Col V is related to autoimmunity in several pathologies including human SSc [16–20]. As expected, the immunization of mice with Col V triggered anti-Col V antibodies at 120 days after the first immunization, but the serological alterations were surprisingly characterized by the detection of ANA, anti-col III, and anti-col IV autoantibodies. Interestingly, the detection of anti-collagen IV autoantibody in the Col V-immunized mice also coincides with the vascular injury that we described since this collagen is present in the basal membrane of the vessels. Furthermore, anti-Col IV antibodies in the serum of human SSc patients and experimental models were previously reported, suggesting that autoantibodies to basement membrane collagen may participate in the pathogenesis of SSc [33, 34].

To gain additional insight into the in vivo tissue injury of the Col V-immunized mice, we investigated the skin and lung changes at morphological and molecular levels. We found that the lung involvement in the Col V-induced SSc mice resembled the fibrotic NSIP, a major histopathological feature of human SSc-associated lung disease. In agreement with the remodeling process characterized by fibrosis and an increase in total collagen content, the lung mechanics exhibited a significant increase in tissue elastance and airway resistance in the immunized animals. Furthermore, a morphological analysis of the skin of the Col V-immunized mice demonstrated an increase of collagen deposition, resulting in fibrosis and skin induration.

To better understand the mechanisms and to identify candidate genes that might be involved in fibrogenesis, we performed qRT-PCR on whole lung and skin mRNA that was obtained from the Col V-immunized mice. The COL1A1, COL1A2, COL3A1, COL5A1, and COL5A2 genes represented a significantly higher expression of Col V in immunized mice, which can reflect the increases in the deposition of Col I and Col V fibers in the lung, suggesting an established fibrotic process. In contrast, only COL5A1 and COL5A2 gene expression was significantly increased in the skin, resulting in the formation of the Col III and Col V fine fibers, also observed in the early stage of SSc in humans [21].

We also found that VEGF, ET-1, and caspase-3 were significantly expressed by endothelial cells in the vascular areas of Col V-immunized mice and may be involved in the complex relationship between vasculopathy and fibrosis in human SSc, as previously demonstrated [35]. In addition, a high expression of VEGF aggravates lung fibrosis and vasculopathy in experimental models of SSc [36] and is also markedly increased in the epidermis and dermis of patients with SSc [37]. Other reports have demonstrated that serum levels of VEGF are significantly increased in SSc patients and show a relationship with the presence of pulmonary arterial hypertension [38–40]. In this context, we hypothesize that the Col V-immunized SSc mouse model can be an important tool to detect events occurring at the endothelial level in the lung and skin. The increased levels of ET-1 and apoptosis by caspase-3 support the notion that injury to the endothelium can increase the levels of vasoconstrictors, as reported in SSc patients [41], and also explains the fibroblast activation and increased deposition of collagen that was observed in this experimental model [42].

We consider that our study has several limitations. The lung phenotype of the Col V mice should be characterized over time to determine whether microvascular disease and inflammation precede lung fibrosis. Finally, mouse models of systemic involvement that also show coexisting vascular alterations and fibrosis such Col V-deficient mice should also be evaluated. In addition, the mechanisms involved in the triggering of SSc in the experimental model of C57BL/6 mice immunized with Col V are not fully understood.

In summary, we have reported that C57BL/6 mice immunized with Col V presented a variety of associated effects found in human SSc, including vasculopathy and autoimmunity with fibrosis of the skin and internal organs, thus emerging as a valuable tool to understand the pathophysiology and to evaluate novel therapeutic approaches in SSc. Further studies are necessary to clarify the action of circulating Col V fragments, anti Col V antibodies, and/or Col V-anti Col V immunocomplexes in the activation of endothelial cells, induction of increased collagen production in organs and autoimmune manifestations.

## Data Availability

The datasets used and/or analyzed during the current study are available from the corresponding author on reasonable request.

## Abbreviations

ANA: Antinuclear antibodies
BM: Basement membrane
BSA: Bovine serum albumin
CASP3: Caspase-3
Col I: Type I collagen
Col V: Type V collagen
COL1A1: Collagen I α1 chain gene
COL1A2: Collagen I α2 chain gene
COL1A3: Collagen III α1 chain gene
COL5A1: Collagen V α1 chain gene
COL5A2: Collagen V α2 chain gene
Col III: Type III collagen
CT: Control
ET-1: Endothelin-1
Gtis: Tissue damping
H&E: Hematoxylin-eosin
HCl: Hydrochloric acid
ICAP: International Consensus on ANA Patterns
IMU-COLV: C57BL/6 Mice immunized with collagen V
NSIP: Nonspecific interstitial pneumonia
OD: Optic density
PBS: Phosphate buffer saline
pNPP: p-Nitrophenyl phosphate
qRT-PCR: Quantitative real-time polymerase chain reaction
Raw: Airway resistance
SSc: Systemic sclerosis
This: Tissue elastance
VEGF: Vascular endothelial growth factor
